# Development, validation and effectiveness of a parents led neuromotor training module with AI-based movement analysis for improving motor proficiency and sports participation in children with developmental coordination disorder: a protocol for an assessor-blinded randomized superiority controlled trial

**DOI:** 10.3389/fnhum.2026.1931622

**Published:** 2026-09-11

**Authors:** Sharath Hullumani, Irshad Qureshi

**Affiliations:** 1 Department of Pediatrics Physiotherapy, Ravi Nair Physiotherapy College, Datta Meghe Institute Higher Education and Research (DU), Wardha, India; 2 Department of Neuro Physiotherapy, Ravi Nair Physiotherapy College, Datta Meghe Institute Higher Education and Research (DU), Wardha, India

**Keywords:** artificial intelligence, DCD, developmental coordiantion disorder, media pipe, neuromotor training, parent led, physical therapy, therapist supervision

## Abstract

**Introduction:**

Developmental Coordination Disorder (DCD) is a common neurodevelopmental condition characterized by impaired motor coordination, poor balance, reduced participation in physical activities, and limitations in functional performance. Conventional rehabilitation often requires prolonged therapist supervision and may not be consistently accessible, particularly in resource-limited settings. Parent-led rehabilitation and artificial intelligence (AI)-based movement analysis have emerged as promising approaches to enhance motor outcomes; however, evidence integrating these strategies remains limited. This manuscript presents the protocol for a randomized controlled trial evaluating a structured Parent-Led Neuromotor Training Programme integrated with AI-based movement analysis in children with DCD.

**Methods and analysis:**

This protocol describes an assessor-blinded, two-arm, parallel-group randomized superiority controlled trial involving 62 children aged 7–14 years diagnosed with DCD according to the Diagnostic and Statistical Manual of Mental Disorders, Fifth Edition (DSM-5) criteria and scoring below the age-expected range on the Movement Assessment Battery for Children–Second Edition (MABC-2). Participants will be randomly allocated (1:1) to either an intervention group receiving a Parent-Led Neuromotor Training Programme integrated with AI-based movement analysis in addition to conventional physiotherapy or a control group receiving conventional physiotherapy alone. Parents will deliver supervised home-based training for 30–45 min per session, 5 days per week, for 12 weeks. Movement analysis will be performed using Google MediaPipe Pose, a validated markerless pose-estimation framework, to quantify joint kinematics, movement symmetry, coordination, movement smoothness, movement accuracy, movement speed, and task-completion time from standardized video recordings. The primary outcome will be motor proficiency, assessed using the Bruininks–Oseretsky Test of Motor Proficiency, Second Edition (BOT-2). Secondary outcomes will include AI-based movement metrics such as symmetry index, movement variability, and coordination score, as well as parental adherence, engagement, satisfaction, balance, and agility outcomes, parental adherence and satisfaction, and AI-derived movement metrics. The sample size was calculated to detect a clinically meaningful between-group difference with 80% power at a 5% significance level. Statistical analyses will follow the intention-to-treat principle, with linear mixed-effects models used to compare repeated measurements between groups. Missing data will be handled using appropriate imputation methods where necessary.

**Clinical Trial Registration:**

https://ctri.nic.in/Clinicaltrials/regtrial.php?trialid=164461&EncHid=98252.11023&modid=1&compid=19 Identifier [CTRI/2026/06/112632].

## Introduction

Developmental motor competence is fundamental for children's participation in activities of daily living, academic performance, recreational activities, and organized sports. Developmental Coordination Disorder (DCD) is one of the most common neurodevelopmental disorders, affecting approximately 5–6% of school-aged children worldwide. According to the Diagnostic and Statistical Manual of Mental Disorders, Fifth Edition (DSM-5), DCD is diagnosed when (A) the acquisition and execution of coordinated motor skills are substantially below that expected for chronological age, (B) motor deficits significantly interfere with activities of daily living, academic achievement, vocational activities, leisure, or play, (C) symptom onset occurs during the early developmental period, and (D) the motor difficulties are not better explained by intellectual disability, visual impairment, or another neurological condition affecting movement (Diagnostic and Statistical Manual of Mental Disorders, 2013; Wilson et al., 2012).

Children with DCD frequently demonstrate impaired balance, poor coordination, delayed motor skill acquisition, reduced muscular fitness, and limitations in functional mobility. These impairments often result in decreased participation in physical activities and sports, reduced self-esteem, social isolation, anxiety, and an increased risk of sedentary behavior and secondary health complications throughout adolescence and adulthood (Smits-Engelsman et al., 2013). Early identification and evidence-based intervention are therefore essential to improve motor competence, participation, and long-term health outcomes.

Conventional rehabilitation for children with DCD is predominantly therapist-led and clinic-based. Although these approaches have demonstrated effectiveness, they often require prolonged therapist supervision, repeated hospital visits, and considerable financial and logistical resources, limiting their accessibility and long-term sustainability, particularly in rural and resource-constrained settings (Zwicker et al., 2013; Blank et al., 2019). In addition, therapist shortages, travel requirements, scheduling difficulties, and reduced adherence to long-term rehabilitation programmes may further compromise treatment effectiveness. Family-centered rehabilitation has therefore emerged as an alternative model that actively involves parents in intervention delivery. Parent-led home-based programmes can increase therapy intensity, promote practice within natural environments, enhance skill generalization, improve adherence, and reduce healthcare costs while empowering families to participate in their child's rehabilitation (Melnyk et al., 2004).

Recent advances in artificial intelligence (AI) have further expanded opportunities for objective movement assessment during rehabilitation. Markerless motion capture systems based on computer vision, such as Google MediaPipe Pose, enable automatic estimation of body landmarks from conventional smartphone videos without requiring wearable sensors or specialized laboratory equipment. These systems can quantify clinically relevant movement parameters including joint kinematics, movement symmetry, coordination, movement smoothness, movement speed, movement variability, movement accuracy, and task completion time, providing objective feedback to therapists and families while supporting remote monitoring of rehabilitation progress (Polatajko and Mandich, 2004; Prinsen et al., 2018). Previous studies have demonstrated the feasibility and validity of AI-based pose estimation for quantitative movement analysis in rehabilitation settings, although evidence in pediatric neurorehabilitation remains relatively limited.

Current evidence suggests that parent-led rehabilitation programmes can improve motor performance, participation, and adherence, while AI-based movement analysis offers objective and scalable assessment of motor function. However, these approaches have largely been investigated independently. Few studies have integrated structured parent-delivered neuromotor training with AI-assisted movement analysis into a comprehensive rehabilitation programme for children with DCD. Furthermore, existing studies have often been limited by small sample sizes, lack of standardized parent training, absence of objective biomechanical outcome measures, limited long-term follow-up, or non-randomized study designs. Consequently, there remains insufficient high-quality evidence regarding the effectiveness of combining family-centered rehabilitation with AI-supported movement analysis to improve motor proficiency and sports participation in children with DCD.

To address these gaps, the present study proposes a structured Parent-Led Neuromotor Training Programme integrated with AI-based movement analysis using Google MediaPipe Pose within an assessor-blinded randomized controlled trial. This integrated approach aims to provide an accessible, family-centered, and technology-assisted rehabilitation model capable of enhancing motor proficiency, objectively monitoring movement quality, and promoting participation in sports and physical activities among children with DCD.

### Objectives

The objectives of this study are:

1. To develop and validate a structured Parent-Led Neuromotor Training Module integrated with AI-based movement analysis for children with Developmental Coordination Disorder.
2. To evaluate within-group changes in motor proficiency, AI-derived movement quality, and sports participation following the 12-week parent-led neuromotor training programme.
3. To compare the effectiveness of the Parent-Led Neuromotor Training Programme integrated with AI-based movement analysis vs. conventional physiotherapy in improving motor proficiency, movement quality, and sports participation among children with Developmental Coordination Disorder.

### Trial design

This study will be conducted as an assessor-blinded, two-arm, parallel-group randomized superiority controlled trial with a 1:1 allocation ratio. The superiority design was selected because the intervention combines structured parent-led neuromotor rehabilitation with AI-based movement analysis, both of which are expected to provide additional clinical benefits beyond conventional physiotherapy. The study will be carried out in the Department of Pediatric Physiotherapy at Datta Meghe Institute of Higher Education & Research, Acharya Vinoba Bhave Rural Hospital. The target population will include children aged 7–14 years diagnosed with Developmental Coordination Disorder. Participants will be recruited using simple random sampling, and allocation will be performed through computer-generated random numbers with concealed allocation. The study will follow assessor blinding to minimize assessment bias. Children fulfilling the inclusion criteria, including diagnosis based on DSM-5 criteria and below age-expected motor performance on standardized assessments such as the Movement Assessment Battery for Children-2 (MABC-2), will be included. Children with neurological disorders, severe intellectual disability, major sensory impairments, or behavioral conditions affecting participation will be excluded. Primary outcome measures will include motor proficiency assessed using standardized tools such as the Bruininks-Oseretsky Test of Motor Proficiency (BOT-2) or MABC-2, along with sports participation measures evaluating the frequency and duration of engagement in physical activities. Secondary outcomes will include AI-based movement metrics such as symmetry index, movement variability, and coordination score, as well as parental adherence, engagement, satisfaction, balance, and agility outcomes. Following ethical approval and trial registration, eligible participants will undergo baseline assessment, after which they will be randomly allocated into intervention and control groups. The intervention group parents will receive structured neuromotor training delivered by physiotherapists focusing on motor skills and sports-related activities. The study module will be implemented in sequential stages including development of the module and validation using the Delphi method prior to the main intervention phase. Parents will be instructed to conduct the training program at home for 30–45 min per session, 5 days per week, for a duration of 12 weeks. Parents in the intervention group will also record videos of their children performing specific motor tasks at regular intervals. These videos will be analyzed using an AI-based movement analysis system employing pose estimation techniques to generate quantitative metrics related to movement quality, coordination, and symmetry. Based on these outputs, individualized feedback will be provided to parents to modify and optimize the training program. The control group will receive standard care, including routine advice regarding physical activity without structured intervention or AI-based monitoring. Outcome assessments will be conducted at baseline, mid-intervention (6 weeks), post-intervention (12 weeks), and follow-up (24 weeks) by blinded assessors. Data collected will include motor proficiency scores, sports participation levels, AI-derived movement metrics, and parental adherence. All data will be systematically recorded, verified, and analyzed using appropriate statistical methods. The effectiveness of the intervention will be determined by comparing changes in outcome.

### AI-based movement analysis system

Movement analysis will be performed using Google MediaPipe Pose, an open-source, real-time, markerless human pose estimation framework capable of detecting 33 anatomical landmarks from RGB video captured using standard smartphone cameras. Parents will record standardized videos using Android or iOS smartphones positioned approximately 2–3 m from the participant under standardized lighting conditions. Videos will be recorded at 30 frames per second with a minimum resolution of 1080 p. The MediaPipe Pose framework (Wagh et al., 2025) employs a two-stage deep learning architecture consisting of person detection followed by pose landmark estimation. Extracted landmark coordinates will be processed using customized Python algorithms to compute objective biomechanical variables including:

- Joint angles
- Range of motion
- Movement symmetry index
- Movement smoothness
- Velocity
- Temporal coordination
- Task completion time
- Inter-limb coordination
- Movement variability

Videos will undergo quality checks before analysis. Poor-quality recordings affected by occlusion, inadequate lighting, incorrect camera positioning, or partial body visibility will be excluded and repeated. Therapists will review AI-generated reports and provide individualized progression recommendations to parents through weekly teleconsultation sessions. MediaPipe Pose has demonstrated excellent validity for markerless motion capture, showing strong agreement with laboratory-based motion analysis systems for joint kinematics and functional movement assessment, making it suitable for remote pediatric rehabilitation.

### AI-based motion capture acquisition protocol

The AI-assisted movement analysis will be performed using Google MediaPipe Pose, an open-source, markerless human pose estimation framework capable of estimating 33 anatomical landmarks from RGB video images. To ensure reproducibility and consistency across participants, all video recordings will follow a standardized acquisition protocol.

Parents will record videos using a smartphone (Android or iOS) equipped with a minimum 1080p (1,920 × 1,080 pixels) camera resolution at 30 frames per second (fps). Recordings will be performed using the rear camera whenever possible to maximize image quality. The camera will be positioned on a stable tripod approximately 2.5–3.0 m from the participant, with the optical axis perpendicular to the frontal plane of the child during frontal tasks and perpendicular to the sagittal plane during side-view assessments. The camera height will be adjusted to approximately the level of the child's pelvis to ensure full-body visualization throughout movement execution.

**Videos will be recorded under standardized environmental conditions, including:**

- Uniform lighting (minimum 300 lux)
- Plain, non-reflective background
- Absence of shadows
- No moving objects within the recording area
- Adequate space (minimum 3 × 3 m)

Participants will wear tight-fitting clothing that clearly exposes major body segments while avoiding loose garments that could obscure anatomical landmarks. Shoes will remain consistent throughout all assessments.

Each participant will perform standardized motor tasks under identical recording conditions at baseline, 6 weeks, 12 weeks, and 24 weeks.

### Technical validation and quality assurance

MediaPipe Pose has demonstrated excellent agreement with laboratory-based marker-based motion capture systems for estimating lower- and upper-limb kinematics in rehabilitation settings. Prior validation studies have reported landmark localization errors generally ranging between 15 and 25 mm, depending on movement complexity, camera positioning, and environmental conditions. To maximize measurement reliability, every recording will undergo an automated and manual quality-control procedure before analysis. Videos will be excluded and reacquired if any of the following occur:

- Less than 95% valid landmark detection
- Major body segment occlusion
- Poor illumination
- Excessive motion blur
- Camera movement during recording
- Participant leaving the camera field
- Landmark tracking loss for more than five consecutive frames

**Movement reliability will be evaluated using:**

- Intraclass Correlation Coefficient (ICC)
- Root Mean Square Error (RMSE)
- Percentage of successfully tracked frames
- Mean landmark confidence score generated by MediaPipe

For repeated movement recordings, ICC values greater than 0.80 will be considered acceptable reliability.

### Signal processing and AI data processing

Raw landmark coordinates obtained from MediaPipe Pose will be exported into Python for further analysis. To reduce measurement noise, all coordinate trajectories will be processed using a fourth-order zero-lag Butterworth low-pass filter with a cut-off frequency of 6 Hz.

Short periods of missing landmark detection (< 5 consecutive frames) will be reconstructed using cubic spline interpolation. Recordings with missing data exceeding 5 consecutive frames or more than 5% of the total recording duration will be excluded from analysis and repeated.

Following preprocessing, two-dimensional joint kinematics and movement descriptors will be automatically calculated using customized algorithms developed in Python.

### Inclusion criteria

- Children aged 7–14 years
- Diagnosed with Developmental Coordination Disorder based on DSM-5 criteria
- Motor performance below age-expected level (e.g., MABC-2 score ≤ 16th percentile)
- Able to understand and follow simple verbal instructions
- Parents/caregivers willing to participate in and deliver the home-based intervention programme
- Children medically stable and able to participate in physical activity and sports-related training.

### Exclusion criteria

- Children with neurological disorders such as cerebral palsy, muscular dystrophy, epilepsy, or other neurodevelopmental conditions affecting movement
- Severe intellectual disability limiting participation in assessment or intervention
- Significant visual or hearing impairments affecting task performance
- Behavioral or psychiatric conditions preventing cooperation during intervention or assessment
- Children currently receiving other structured motor rehabilitation programmes
- Any recent musculoskeletal injury or medical condition contraindicating physical activity participation.

### Operational framework of the intervention

The intervention will be delivered over 12 weeks, 5 days per week, for ~30–45 min per session. It will follow a standardized phased framework consisting of parent preparation, foundational neuromotor training, progressive task-oriented training, sport-specific skill development and consolidation. All intervention families will receive the same core programme. Individualization will be restricted to predefined progression or regression criteria based on the child's performance, safety, adherence and AI-derived movement feedback.

### Participant timeline

Participants will undergo screening, baseline assessment, randomization, 12 weeks of intervention/control management with mid-intervention assessment at 6 weeks, post-intervention assessment at 12 weeks, and follow-up evaluation at 24 weeks and elicited in Table 1.

**Table 1:** Participant timeline.

Phase	Weeks	Main focus	Intervention components
Parent preparation	Before Week 1	Parent competency and safety	DCD education, demonstration of activities, video-recording training, safety procedures and competency assessment
Familiarization	1–2	Basic movement control	Postural control, balance, bilateral coordination, simple gross- and fine-motor tasks and introductory action observation
Skill acquisition	3–5	Neuromotor learning	Dynamic balance, agility, hand–eye coordination, locomotor tasks and graded motor imagery
Skill progression	6–8	Increased task complexity	Multi-step motor tasks, direction changes, dual-component activities, ball handling and AI-guided correction
Sports integration	9–10	Sport-related participation	Throwing, catching, kicking, running drills, target activities and modified sport-specific tasks
Consolidation	11–12	Functional transfer and independence	Practice of individualized target activities, home/community transfer, reduced prompting and independent task performance
Retention period	13–24	Maintenance	Continued routine activity advice, adherence monitoring and follow-up assessment without supervised progression

### Group A

Participants allocated to Group A will receive a structured Parent-Led Neuromotor Training Programme integrated with AI-Based Movement Analysis along with conventional physiotherapy management. The intervention will be designed to improve motor proficiency, coordination, balance, agility, functional performance, and sports participation in children diagnosed with Developmental Coordination Disorder. The programme will follow a systematic multi-stage approach to ensure scientific validity, clinical applicability, feasibility, and scalability for home and community-based implementation.

The complete SMART-MOVE DCD intervention manual, including detailed descriptions of parent education, neuromotor training, action observation and motor imagery, sport-specific activities, AI-based movement analysis procedures, progression criteria, intervention dosage, therapist instructions, parent competency assessment, fidelity monitoring, and home implementation procedures, is provided in Supplementary File 1. The supplementary document has been prepared in accordance with the Template for Intervention Description and Replication (TIDieR) checklist to facilitate accurate replication by clinicians and researchers.

**The intervention module will consist of three major stages:**

(1) Development and Validation of the Module (Table 2 and Figure 1)
(2) Sensitisation and Standardization,
(3) Pilot study

**Table 2:** Triple ‘O' model.

Objectives	Operations	Expected Outcomes
• To develop and validate a structured Parent-Led Neuromotor Training Module for children with Developmental Coordination Disorder through literature review, evidence synthesis, focused group discussions, Delphi validation, and standardization procedures. • To establish a scientifically rigorous, clinically feasible, and home-based rehabilitation framework integrating parent participation and AI-supported rehabilitation strategies.	• Development of the intervention framework through literature review, focused group discussions, module structuring, Delphi expert validation, pilot testing, and standardization. • Implementation of parent education, neuromotor and sports-specific training, home-based intervention sessions, tele-supervision, and AI-based movement monitoring using pose estimation techniques.	• Development of a validated, standardized, and clinically applicable rehabilitation module suitable for home and community settings. • Improved feasibility, scalability, parental adherence, and integration of AI-assisted rehabilitation into pediatric physiotherapy practice.
• To improve motor proficiency, coordination, balance, agility, and sports participation in children with DCD using parent-led neuromotor and sports-specific training programmes. • To evaluate the effectiveness of AI-based movement analysis and parent-mediated intervention compared with conventional physiotherapy.	• Delivery of structured balance, coordination, agility, fine motor, and sports-related functional training combined with AI-assisted movement analysis and blinded outcome assessment. • Randomized controlled trial implementation with baseline, mid-intervention, post-intervention, and follow-up evaluations using standardized assessment tools and statistical analysis.	• Improvement in BOT-2/MABC-2 scores, sports participation levels, movement symmetry, coordination, balance, and functional performance. • Generation of evidence supporting the effectiveness of AI-assisted parent-led rehabilitation programmes for children with DCD.

**Figure 1:**
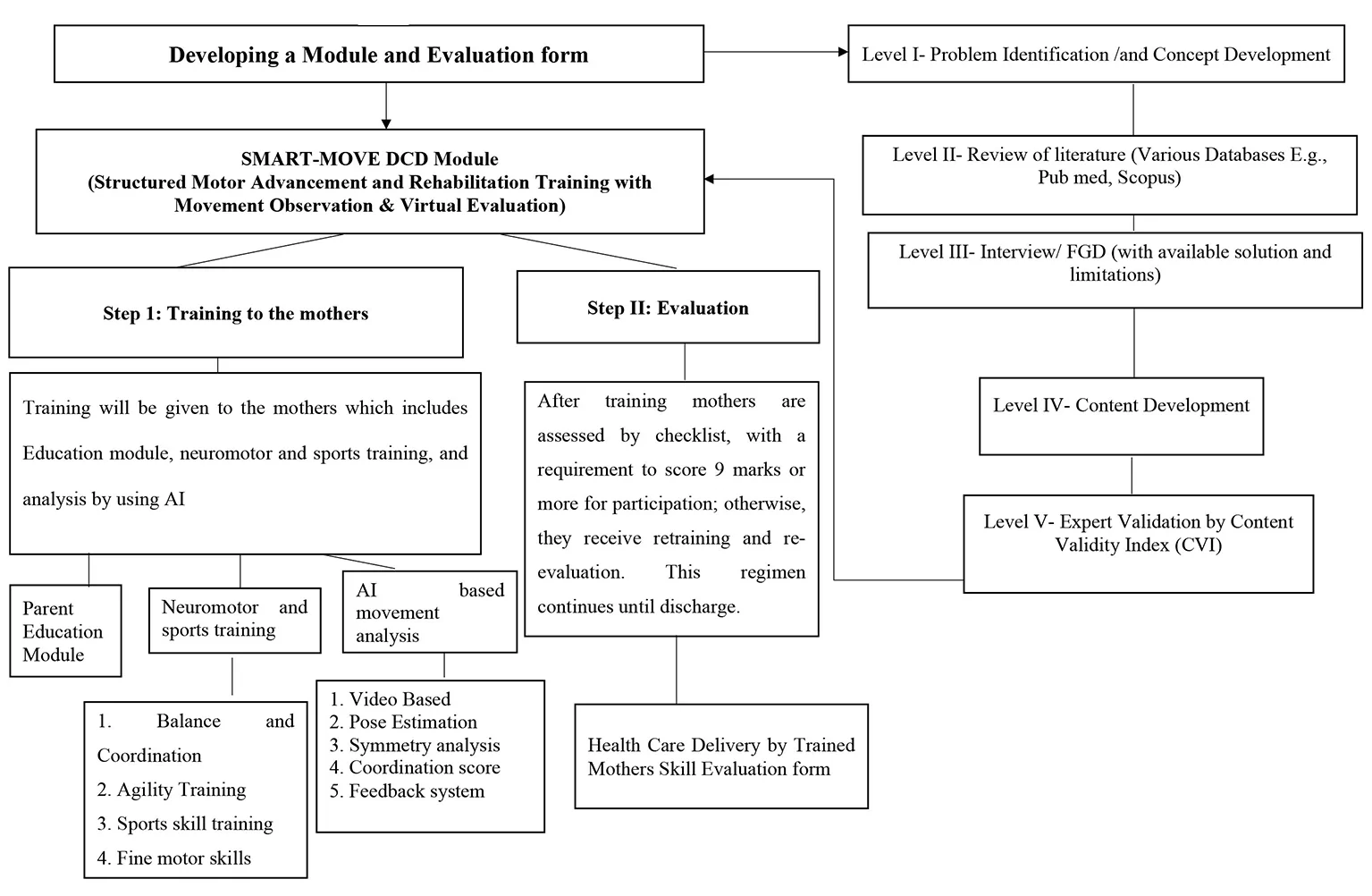
Step 2-Operations of Triple ‘O' model (Hullumani and Qureshi, 2026; Qureshi et al., 2024).

The development process will be guided by the Hullumani, Qureshi Framework for Development and Validation of Clinical Protocols, Educational Modules, and Questionnaires in Healthcare Research (Figure 2).

**Figure 2:**
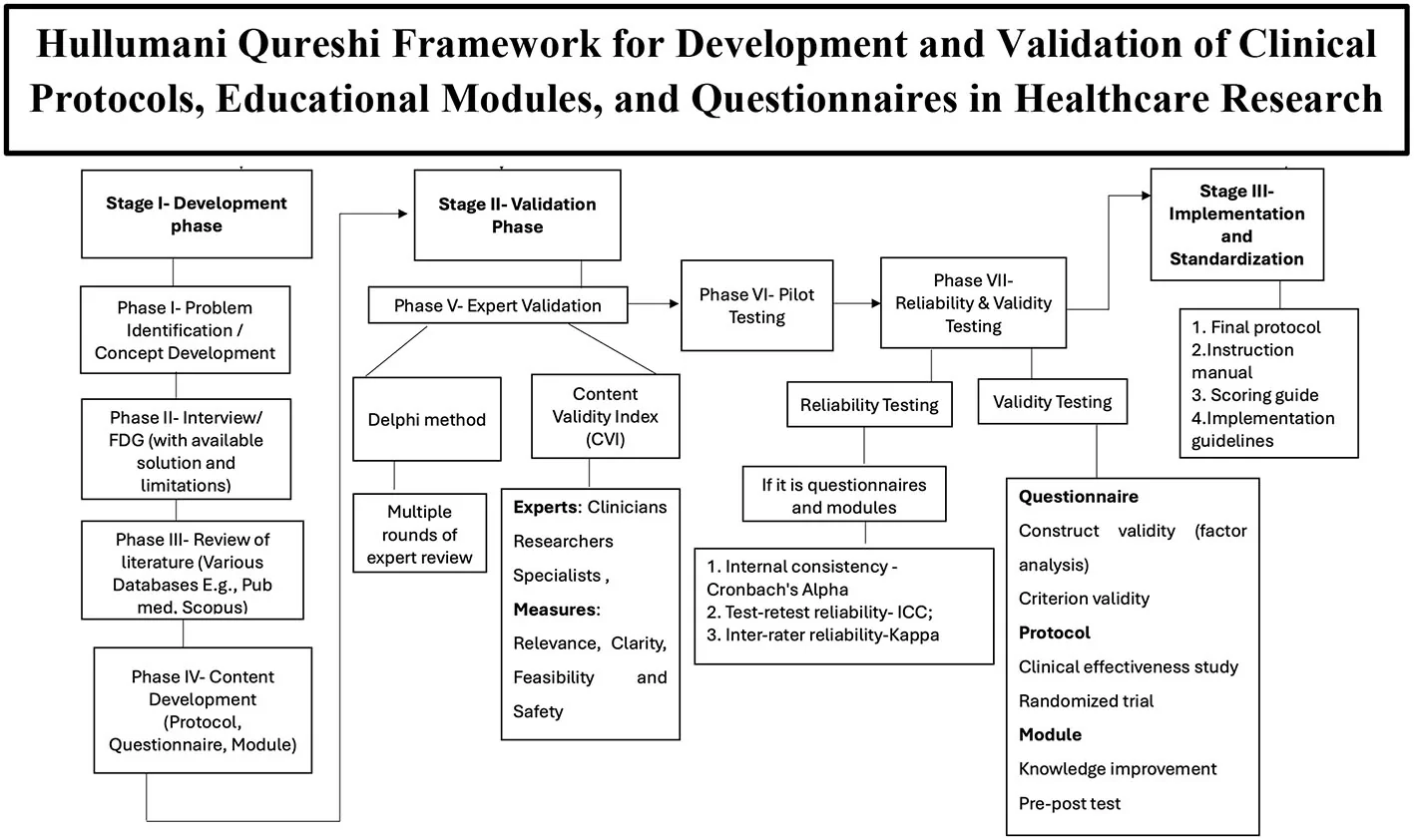
Hullumani, Qureshi framework for development and validation of clinical protocols, educational modules, and questionnaires in healthcare research.

The module will be developed using focused group discussions, evidence synthesis, and a structured framework approach to ensure comprehensive integration of motor learning principles, rehabilitation strategies, sports participation concepts, and technology-assisted movement analysis.

### Stage I: development of the module

The development and validation of the training module and skill assessment tool were conducted in accordance with COSMIN guideline (Prinsen et al., 2018). Further, the module was conceptually inspired by and academically adapted from the Triple ‘O' Model developed (Qureshi et al., 2024) by Dr Gaurav V Mishra (Copyright Registration No. L-99015/2021). Formal permission for academic utilization and adaptation of the model was obtained from the author.

The Triple “O” model in educational research, meaning the Objectives, Operations, and Outcomes model, is a plan to dive into how research works (Table 2).

- Objectives are the reasons for this research, directing what researchers aim to explore.
- Operations focus on transforming these reasons into actual steps; they navigate the methods for gathering information.
- As for the Outcomes, they represent significant shifts resulting from the research, such as improved methods of teaching or students achieving their educational targets with better learning outcomes.

The first stage will focus on systematic development of the Parent-Led Neuromotor Training Programme with AI-Based Movement Analysis. Initially, an extensive needs assessment and conceptualization process will be carried out through review of current literature on developmental coordination disorder, analysis of existing neuromotor rehabilitation approaches, sports participation interventions, and identification of gaps in parent-led rehabilitation models. This phase will highlight limitations such as restricted access to therapist-led interventions, inadequate long-term home programmes, poor integration of sports training into rehabilitation, and limited utilization of artificial intelligence in pediatric motor rehabilitation.

### Standard session structure

Each 30–45-min home session will include:

1. **Warm-up:** 5 min Mobility exercises, light locomotor activity and preparation for task performance.
2. **Action observation and motor imagery:** 5 min The child will observe a standardized demonstration and mentally rehearse selected movements before physical practice.
3. **Neuromotor training:** 15–20 min Balance, postural control, bilateral coordination, agility, gross-motor and fine-motor activities.
4. **Sport-specific or participation-focused practice:** 10–15 min Throwing, catching, kicking, running, target tasks or individualized participation goals.
5. **Cool-down and documentation:** 5 min Gentle activity, symptom check, parent logbook completion and recording of adherence or adverse events.

### Parent competency assessment and certification

Before initiating the home-based intervention, each participating parent or caregiver allocated to the intervention group will complete a structured training programme delivered by a pediatric physiotherapist. Training will include explanation, therapist demonstration, guided practice, return demonstration, video-recording procedures, exercise progression, safety monitoring, documentation, and data-privacy requirements.

Parental competency will be evaluated using a 12-item Parent Competency Assessment Checklist developed specifically for the intervention. The checklist evaluates the parent's ability to prepare a safe environment, position the child, demonstrate the prescribed balance, coordination, task-oriented, and sports-related activities, provide appropriate instructions and feedback, follow exercise dosage and progression criteria, identify adverse responses, record standardized videos, document adherence, and comply with safety and privacy procedures.

Each item will be scored as 0, 1, or 2, where 0 indicates inability or incorrect performance, 1 indicates partial performance or performance requiring prompting, and 2 indicates correct and independent performance. The maximum score will be 24. Parents will be required to obtain a minimum score of 22 out of 24 and achieve the maximum score on all safety-critical items before being certified to conduct the intervention independently. Parents who do not achieve the threshold will receive additional training followed by reassessment. Home-based training will commence only after the competency criterion has been fulfilled.

### Validation of the parent competency checklist

Two trained pediatric physiotherapists will independently assess parental performance during a standardized return-demonstration session. Content validity of the checklist will be evaluated through a two-round Delphi process using the Item-Level and Scale-Level Content Validity Indices. Inter-rater reliability will be assessed during pilot testing using ICC (2,1), with values of at least 0.75 considered acceptable. The complete checklist, scoring instructions, and certification criteria are provided in Supplementary File 2.

Content validity will be quantified using:

- Item-Level Content Validity Index (I-CVI)
- Scale-Level Content Validity Index (S-CVI/Ave)

An **I-CVI** **≥0.78** and **S-CVI** **≥0.90** will be considered indicative of excellent content validity.

Following content validation, the checklist will be pilot tested in a sample of parents not participating in the main trial. Inter-rater reliability between two independent pediatric physiotherapists will be evaluated using the Intraclass Correlation Coefficient.

Interpretation of ICC:

- ICC ≥0.90 = Excellent reliability
- ICC 0.75–0.89 = Good reliability
- ICC 0.50–0.74 = Moderate reliability
- ICC < 0.50 = Poor reliability

The finalized checklist will be incorporated into the intervention manual before commencement of the randomized trial.

### Exercise dosage and progression

Each activity will be documented in an intervention manual specifying:

- starting position;
- equipment required;
- number of repetitions;
- number of sets;
- rest interval;
- task duration;
- verbal instructions;
- assistance permitted;
- safety precautions;
- progression and regression criteria.

**An activity will be progressed when the child:**

- completes at least 80% of trials correctly;
- maintains appropriate movement quality;
- performs the task without unsafe compensatory movement;
- reports no pain, excessive fatigue or distress;
- demonstrates the required performance across two consecutive sessions.

**Progression include:**

- increasing repetitions or duration;
- reducing the base of support;
- increasing movement speed;
- increasing target distance;
- adding directional changes;
- decreasing external assistance;
- introducing dual-task demands;
- increasing sport-specific complexity.

Tasks will be regressed when the child cannot complete at least 60% of attempts correctly, demonstrates repeated loss of balance, reports discomfort or exhibits substantial compensatory movement.

### Integration of AI-based feedback

Standardized videos will be submitted once weekly during the intervention. AI-based analysis will be performed for predefined tasks rather than every exercise.

The AI output will be used to identify:

- asymmetry;
- excessive movement variability;
- reduced movement smoothness;
- inaccurate joint positioning;
- delayed task completion;
- coordination errors.

A physiotherapist will review the AI-generated outputs before communicating recommendations. The AI system will support, but will not independently prescribe, progression (Table 3).

**Table 3:** Intervention modifications will be selected from a predefined decision framework.

AI finding	Standardized response
Persistent bilateral asymmetry	Reduce task complexity and emphasize symmetrical practice
Reduced movement accuracy	Provide visual demonstration and external target cues
Excessive variability	Increase repeated practice at a slower speed
Reduced smoothness	Break the task into components and gradually combine them
Adequate performance across two submissions	Progress task difficulty according to the manual
Tracking failure or poor recording	Repeat the recording; do not use the affected output for progression

### Intervention fidelity and adherence

Intervention fidelity will be monitored through:

- parent training and competency assessment;
- standardized intervention manuals;
- daily parent logbooks;
- weekly video submissions;
- weekly telephone or video supervision;
- periodic review of a random sample of intervention sessions;
- documentation of exercise progression and protocol deviations.

**Adherence will be calculated as:**


$$\begin{array}{l} \text{Adherence (\%)}=\frac{\text{Number of completed sessions}}{\text{Number of prescribed sessions}}\times 100 \end{array}$$

Completion of at least 80% of the prescribed sessions will be considered acceptable adherence. However, all randomized participants will remain included in the intention-to-treat analysis irrespective of adherence.

### Parent training and intervention fidelity

Prior to the initiation of the intervention, all parents allocated to the intervention group will participate in a standardized competency-based training programme conducted by experienced pediatric physiotherapists. The training programme is designed to ensure that every parent acquires the minimum level of knowledge and practical competency required to safely and consistently administer the home-based neuromotor training programme. The training will include education regarding Developmental Coordination Disorder (DCD), its clinical characteristics, functional implications, and the rationale for the intervention. Parents will receive detailed demonstrations of each intervention activity, followed by supervised hands-on practice sessions to ensure correct execution of the exercises. Additional training components will include instruction on safe handling techniques, appropriate exercise progression, standardized AI-based movement recording procedures, home safety precautions, and proper documentation of daily home practice.

Upon successful completion of the training programme, each parent will receive a standardized intervention package comprising a printed parent manual, an illustrated exercise guide, QR code-linked instructional videos demonstrating each exercise, an AI recording guide outlining camera positioning and recording procedures, and a structured logbook for documenting daily intervention activities. The competency of each parent will be verified before independent implementation of the intervention to ensure that all participants possess the required skills to deliver the programme safely and effectively.

To minimize variability and maintain consistency throughout the trial, all parents will receive identical intervention materials and standardized instructions. Every participant will be provided with the same parent manual, identical exercise dosage and frequency, standardized instructional videos, uniform progression criteria, and the same equipment list. Parents will be instructed not to modify, replace, or progress any exercise independently without prior approval from the supervising pediatric physiotherapist. Any modification to the intervention protocol will only be implemented following clinical review to ensure uniformity across all participants.

Intervention fidelity will be monitored throughout the study using multiple complementary strategies. Weekly teleconsultation sessions will be conducted to reinforce correct exercise techniques, address parental concerns, and encourage adherence to the intervention protocol. AI-generated movement recordings submitted by parents will be reviewed weekly to verify correct exercise performance and recording quality. Parents will also maintain weekly exercise logbooks documenting the completion, duration, and any challenges encountered during home practice. Standardized video recordings of intervention sessions will be submitted every week for therapist evaluation, while random therapist observations will be conducted periodically to verify protocol adherence. In addition, monthly fidelity audits will be performed using a predefined fidelity checklist to identify protocol deviations and provide corrective feedback whenever necessary.

A standardized fidelity checklist will be completed weekly by the supervising pediatric physiotherapist for every participant. The checklist will evaluate whether the exercises were performed correctly, the prescribed dosage was maintained, safety precautions were followed, AI-recorded videos met the required quality standards, parents adhered to the intervention protocol, children actively participated in the sessions, and whether any protocol deviations occurred. Each item will be recorded as either **“Yes”** or **“No”** to provide a structured assessment of intervention fidelity (Table 4).

**Table 4:** Fidelity checklist.

Item	Yes	No
Exercise completed correctly	□	□
Correct dosage	□	□
Safety maintained	□	□
AI video acceptable	□	□
Parent followed protocol	□	□
Child engaged	□	□
No deviations	□	□

Each week therapists will complete,

Participant adherence to the intervention will be quantified by calculating the percentage of completed sessions relative to the total number of prescribed sessions using the formula: **Adherence (%)** **=** **(Completed Sessions/Prescribed Sessions)** **×** **100**. Adherence will subsequently be categorized as **excellent (≥90%)**, **good (80–89%)**, **moderate (60–79%)**, or **poor (< 60%)**. These categories will facilitate objective monitoring of parental compliance throughout the intervention period.

Adherence will be calculated as,


$$\begin{array}{l} \frac{\text{Completed Sessions}}{\text{Prescribed Sessions}}\times 100 \end{array}$$

Protocol deviations will be predefined and systematically documented throughout the study. Deviations will include missing more than 20% of the prescribed intervention sessions, altering the prescribed order of exercises, skipping the recommended exercise progression, performing incorrect AI recording procedures, introducing unauthorized exercises, failing to submit the required weekly video recordings, or violating established safety procedures during intervention delivery. All protocol deviations will be recorded, reviewed by the study investigators, and managed according to the study protocol to maintain intervention integrity and ensure methodological rigor.

### Retraining

Parents demonstrating poor fidelity (< 80%) during two consecutive weeks will receive individualized retraining before continuing the intervention.

### Therapist standardization

Before recruitment, all physiotherapists involved in the study will undergo standardized training on intervention delivery, outcome assessment, AI-based movement analysis, parent education, and fidelity monitoring. A written intervention manual and competency checklist will be used to ensure uniform implementation across therapists.

### Control-group standardization

The description of the control condition must also be standardized. “Conventional physiotherapy” should not remain undefined. Participants allocated to the control group will receive conventional physiotherapy according to a standardized treatment schedule matched, as far as feasible, for treatment duration and contact frequency. The control intervention includes balance, coordination, strengthening, functional mobility and general physical-activity training but will not include the structured parent-led module, action-observation and motor-imagery package, AI-based movement analysis or AI-informed feedback. Treatment content, duration, attendance and co-interventions will be documented for each participant.

Based on the identified needs, the intervention module will be designed and structured into five major components:

1. **Parent Education Module**: Parents will receive structured education regarding developmental coordination disorder, its functional implications, motor difficulties, psychosocial challenges, and the importance of early intervention. Training will also include principles of motor learning such as repetition, task specificity, feedback, graded progression, and reinforcement strategies. Parents will be trained to function as primary facilitators of the rehabilitation programme within the home environment.
2. **Neuromotor and Sports Training Module**: This component will include exercises targeting gross motor skills, balance, coordination, agility, locomotor abilities, postural control, and functional movement patterns. Fine motor training activities focusing on dexterity, hand control, and hand–eye coordination will also be incorporated. Sports-specific skill training including throwing, catching, running drills, ball control, and functional activity integration will be progressively graded according to the child's abilities and performance.
3. **Action Observation and Motor Imagery (AOMI)**: Action observation and motor imagery techniques will be incorporated to enhance motor learning and skill acquisition. Children will observe therapist-modeled or video-based demonstrations of motor tasks before performing them. This process aims to reinforce movement planning, improve coordination, and facilitate neuroplasticity.
4. **AI-Based Movement Analysis Integration:** The intervention will integrate AI-based movement analysis using Google MediaPipe Pose, an open-source, markerless pose estimation framework. Parents will periodically record standardized videos of their child performing predefined motor tasks using an Android or iOS smartphone. The recorded videos will be analyzed using MediaPipe Pose to estimate body landmarks and generate objective movement quality parameters, including movement symmetry, coordination, movement variability, movement speed, joint alignment, range of motion, and movement accuracy. The generated reports will enable therapists to remotely monitor motor performance, objectively track progress, identify movement deviations, and provide individualized feedback to parents to optimize the home-based rehabilitation programme (Wagh et al., 2025).
5. **Home-Based Implementation and Monitoring**: The programme will be implemented as a structured 12-week home-based intervention. Parents will conduct supervised training sessions for approximately 30–45 min per day, 5 days per week. Compliance and adherence will be monitored through session logbooks, weekly video submissions, tele-supervision, and periodic therapist review sessions. Continuous monitoring will help ensure safety, progression, and consistency of intervention delivery.

### Stage II: validation of the module

The developed intervention module will undergo content validation using the Delphi method. A panel of experts including pediatric physiotherapists, neurodevelopmental specialists, sports rehabilitation experts, and AI/biomechanics professionals will review the module for relevance, clarity, feasibility, clinical applicability, and comprehensiveness. Suggestions and modifications provided by experts will be systematically incorporated to improve the intervention content and implementation process.

The content of the module will be developed based on evidence-based physiotherapy protocols, pediatric motor learning principles, task-oriented training approaches, activity-based rehabilitation models, and sports skill acquisition frameworks. Exercises will be selected and graded across domains including balance, postural control, coordination, agility, fine motor skills, dexterity, hand–eye coordination, and sports-related activities. Each intervention activity will include standardized instructions, dosage parameters, progression criteria, safety precautions, and therapist guidance to ensure consistency across participants.

Sensitisation sessions will also be conducted for parents and therapists to ensure uniform understanding and delivery of the intervention procedures. Parents will be trained regarding exercise execution, monitoring strategies, video recording procedures, and safety precautions for home implementation.

### Stage III: implementation to the main study (pilot RCT)

Following validation and standardization, the finalized intervention module will be implemented in the main randomized controlled trial. Participants allocated to Group A will undergo the 12-week parent-led neuromotor training programme under physiotherapist supervision. The intervention will combine conventional physiotherapy management with structured home-based neuromotor and sports training supported by AI-based movement monitoring.

The finalized module will include structured session formats, defined progression levels, integrated AI-feedback systems, monitoring protocols, and adherence strategies to ensure reproducibility and scalability. Outcome measures including motor proficiency, sports participation, AI-derived movement metrics, balance, agility, and parental adherence will be assessed at baseline, mid-intervention, post-intervention, and follow-up stages by blinded assessors. The programme is expected to provide an accessible, family-centered, and technology-supported rehabilitation approach capable of improving functional performance and sports participation in children with developmental coordination disorder within home and community settings.

### Outcome measures

#### Primary outcomes

1. Bruininks–Oseretsky Test of Motor Proficiency – Second Edition (BOT-2): A standardized assessment tool used to evaluate fine and gross motor skills, coordination, balance, strength, and agility in children.

#### Secondary outcomes

1. AI-Based Movement Analysis Outcomes by using Google Media pipe,

i Movement Symmetry Index (MSI)

**Purpose**: Evaluates bilateral symmetry of lower- and upper-limb movements during standardized functional tasks.

### Anatomical landmarks

- Right Shoulder (12)
- Left Shoulder (11)
- Right Hip (24)
- Left Hip (23)
- Right Knee (26)
- Left Knee (25)
- Right Ankle (28)
- Left Ankle (27)

(MediaPipe landmark numbering).

#### Pre-processing

- Landmark coordinates normalized to pelvic width
- Butterworth filtering
- Coordinate transformation to body-centered coordinate system

**Formula**


$$\begin{array}{l} MSI=\left(1-\frac{|R-L|}{R+L}\right)\times 100 \end{array}$$

Where,

R = right limb movement amplitude

L = left limb movement amplitude

**Units**- Percentage (%)

**Expected physiological range**- 95–100%

#### Clinical interpretation

Lower values indicate asymmetrical motor execution.

Children with DCD commonly demonstrate:

- unequal step length
- asymmetric reaching
- poor weight transfer

Improvement following intervention is expected to increase MSI toward 100%.

ii Coordination Index

**Purpose:** Quantifies temporal coordination between body segments.

#### Landmarks

- Shoulder
- Hip
- Knee
- Ankle

**Calculation**

Cross-correlation coefficient between adjacent joint trajectories.


$$CI=\frac{\sum \left(x_{i}-x\right)\left(y_{i}-y\right)}{\sigma_{x}\sigma_{y}}$$

**Range**

−1 to +1

iii Movement Accuracy

**Purpose**: Measures deviation from therapist reference movement.

**Landmarks**

All task-specific joints.

**Formula**

Mean Absolute Joint Angle Error


$$\begin{array}{l} MAE=\frac{1}{n}\sum |\theta_{reference}-\theta_{observed}| \end{array}$$

iv Movement Smoothness

Calculated using,

- Spectral Arc Length (SPARC)
- Normalized Jerk Score

Formula


$$\begin{array}{l} Jerk=\frac{d^{3}x}{dt^{3}} \end{array}$$

Higher SPARC
Lower jerk =Smoother movement.
Children with DCD generally show increased jerk and fragmented movement trajectories.

v Movement Speed

Average center-of-mass velocity.

Formula:


$$\begin{array}{l} Velocity=\frac{Distance}{Time} \end{array}$$

Units: m/s
Age-specific normative values.
DCD children usually exhibit slower movement speed due to impaired motor planning.

vi Movement Variability

Calculated as,

Standard deviation of repeated movement trajectories.

Lower variability = Improved motor consistency.

vii Joint Kinematics

Calculated for,

- Hip
- Knee
- Ankle
- Shoulder
- Elbow
- Trunk

Joint angles calculated using,


$$\begin{array}{l} \theta =\cos^{-1}\left(\frac{AB\cdot BC}{|AB||BC|}\right) \end{array}$$

Units: Degrees

viii Task Completion Time

Seconds required to complete,

- balance task
- agility task
- throwing task
- catching task

Lower values indicate improved functional motor performance and overall is mentioned in Table 5.

- Pediatric Balance Scale (PBS): Assesses functional balance performance in children.
- *T*-Test Agility Assessment: Evaluates agility, speed, and directional movement ability.
- Shuttle Run Test: Assesses agility, coordination, and functional mobility.
- Movement Assessment Battery for Children – Second Edition (MABC-2): A validated assessment used to identify and measure motor coordination difficulties and functional motor performance in children.
- Children's Assessment of Participation and Enjoyment (CAPE): A questionnaire designed to assess children's participation, diversity, frequency, and enjoyment in physical, recreational, and social activities

**Table 5:** Summary.

Outcome	Formula	Unit	Healthy range	Expected change
Symmetry index	Bilateral ratio	%	95–100	Increase
Coordination index	Cross correlation	0–1	>0.85	Increase
Movement accuracy	Mean absolute error	Degrees	< 5°	Decrease
Smoothness	SPARC/Jerk	Unitless	Higher	Increase
Speed	Distance/time	m/s	Age norms	Increase
Variability	SD	mm	Low	Decrease
Joint angle	Vector angle	Degrees	Task specific	Normalize
Task time	Stopwatch	Seconds	Lower	Decrease

The primary outcome of the trial will be motor proficiency assessed using the BOT-2 total motor composite score. The primary endpoint will be the adjusted between-group difference in change from baseline to 12 weeks. The MABC-2 will be used for eligibility confirmation and as a secondary motor-coordination outcome. Sports participation, balance, agility, physical fitness, parental outcomes, adherence, intervention fidelity, and AI-derived movement variables will be treated as secondary or exploratory outcomes.

#### Sample size

Reference article: Wilson et al. (2012).

The sample size will be calculated based on the study's single primary outcome: the change in the Bruininks–Oseretsky Test of Motor Proficiency–Second Edition Total Motor Composite score from baseline to 12 weeks.

The calculation will be performed using G^*^Power software for a two-group superiority comparison with equal allocation. The following parameters will be specified:

- Statistical test: Difference between two independent group means
- Effect size: Cohen's *d*
- Type I error: α = 0.05
- Statistical power: 80%
- Allocation ratio: 1:1
- Direction of testing: Two-sided

The expected effect size will be derived from a published randomized or controlled intervention study evaluating a comparable task-oriented, parent-mediated or home-based motor intervention in children with Developmental Coordination Disorder. If an adequately comparable published estimate is unavailable, the effect size will be obtained from pilot-study data and interpreted cautiously.

Using a standardized effect size of **Cohen's** ***d*** **=** **0.70**, a two-sided α of 0.05 and 80% power, the calculation requires approximately **31 participants per group**, resulting in 62 evaluable participants.

## Statistical analysis

Statistical analyses will be performed using IBM SPSS Statistics version 31.0. The distribution of continuous variables will be examined using the Shapiro–Wilk test, histograms, and Q–Q plots. Baseline demographic and clinical characteristics will be summarized using descriptive statistics. Continuous variables will be reported as mean and standard deviation or median and interquartile range, depending on their distribution, whereas categorical variables will be presented as frequencies and percentages.

The primary analysis will follow the intention-to-treat principle, whereby all randomized participants will be analyzed in the groups to which they were originally allocated. A supplementary per-protocol analysis will be conducted among participants who complete the intervention and meet the predefined adherence criteria.

The primary effectiveness analysis will examine the group × time interaction for the BOT-2 total motor composite score, with particular emphasis on the adjusted between-group difference in change from baseline to 12 weeks. Analyses of the MABC-2 and all remaining outcomes will be considered secondary.

Changes in outcomes measured at baseline, 6 weeks, 12 weeks, and 24 weeks will be analyzed using linear mixed-effects models. The models will include fixed effects for treatment group, time, and the group × time interaction, with a participant-specific random intercept to account for within-participant correlations arising from repeated measurements. Baseline outcome values and relevant prespecified covariates will be included where appropriate. The principal treatment effect will be determined from the group × time interaction, particularly the adjusted between-group difference at 12 weeks and 24 weeks.

Model assumptions, including normality and homoscedasticity of residuals, will be assessed. Appropriate transformations, robust estimation procedures, or generalized mixed-effects models will be used where assumptions are not satisfied. Adjusted mean differences will be reported with 95% confidence intervals, corresponding *p*-values, and standardized effect sizes, including Cohen's *d*, where appropriate.

Linear mixed-effects modeling will permit the inclusion of participants with incomplete repeated measurements under the missing-at-random assumption. The amount, pattern, and reasons for missing data will be reported. Where the proportion of missing data is substantial, multiple imputation will be performed as a sensitivity analysis. Additional sensitivity analyses will compare intention-to-treat, per-protocol, and complete-case findings.

The BOT-2 total motor composite score will be treated as the primary outcome. Secondary outcomes will include MABC-2, CAPE, balance, agility, parental outcomes, and AI-derived movement parameters. To reduce the risk of type I error arising from multiple secondary comparisons, *p*-values will be adjusted using an appropriate procedure, such as the Benjamini–Hochberg false discovery rate method. All statistical tests will be two-sided, and statistical significance for the primary outcome will be set at *p* < 0.05.

## Methods: monitoring

### Data monitoring

The study data will be monitored by the Data Monitoring Committee of Ravi Nair Physiotherapy College to ensure accuracy, completeness, protocol adherence, and participant safety throughout the trial period.

### Harms

Any adverse event or unintended effect occurring during the study period will be documented and immediately reported to the Institutional Ethics Committee and the clinician responsible for participant management. Appropriate medical care and monitoring will be provided for all solicited and spontaneous adverse events related to the trial interventions or study procedures.

### Confidentiality

Confidentiality of all participants enrolled in the study will be strictly maintained. Personal and patient-related information will be coded and securely stored, and such information will only be accessed with the participant's and parent's informed permission. Data collected during the study will be used solely for research and academic purposes.

### Declaration of interests

The investigators declare that there are no financial conflicts of interest, competing interests, or external influences associated with this study.

### Access to data

All data collected during and after the study will be securely maintained by the Principal Investigator (PI). The PI will have access to the final trial dataset. De-identified data may be shared upon formal request strictly for academic research and publication purposes.

### Ancillary and post-trial care

Appropriate care and support will be provided to study participants in the event of any harm or adverse effect resulting from trial participation, in accordance with the policies of Ravi Nair Physiotherapy College and Datta Meghe Institute of Higher Education & Research.

### Dissemination policy

The findings of the study will be disseminated through academic and research activities including presentations at national and international conferences and publication in peer-reviewed scientific journals. Data generated from the study will be used exclusively for educational, scientific, and research-related purposes.

## Discussion

Developmental Coordination Disorder (DCD) is one of the most common neurodevelopmental disorders, affecting approximately 5–6% of school-aged children and substantially influencing motor coordination, balance, functional mobility, physical activity participation, and quality of life. Children with DCD frequently experience persistent motor impairments that limit participation in sports, recreational activities, and everyday tasks, often leading to reduced self-confidence, social isolation, and long-term sedentary behavior. Previous studies have demonstrated that task-oriented interventions, motor learning approaches, and parent-supported rehabilitation can improve motor performance and participation; however, access to prolonged therapist-supervised rehabilitation remains challenging because of workforce shortages, travel requirements, financial costs, and limited availability of specialized pediatric rehabilitation services, particularly in low-resource and rural settings (Diagnostic and Statistical Manual of Mental Disorders, 2013; Wilson et al., 2012; Smits-Engelsman et al., 2013; Zwicker et al., 2013; Blank et al., 2019). These limitations highlight the need for accessible, family-centered, and technology-assisted rehabilitation models that extend therapy into the child's natural environment.

The present protocol proposes an assessor-blinded randomized superiority controlled trial evaluating a Parent-Led Neuromotor Training Programme integrated with AI-based movement analysis. A superiority design was selected because the intervention incorporates additional therapeutic components beyond conventional physiotherapy, including structured parent-mediated rehabilitation, action observation, motor imagery, AI-assisted movement monitoring, and individualized feedback, all of which are hypothesized hypothesized to produce superior improvements in motor proficiency and sports participation compared with conventional physiotherapy alone.

Previous investigations have shown that parent-mediated and home-based rehabilitation programmes can improve therapy adherence, increase practice intensity, enhance family engagement, and facilitate motor-skill generalization in children with neurodevelopmental disorders. Likewise, recent studies have demonstrated the feasibility of markerless motion-capture technologies and AI-based pose-estimation systems for objectively quantifying movement characteristics during rehabilitation. Nevertheless, these approaches have largely been investigated independently, and there remains limited evidence integrating structured parent-delivered neuromotor training with AI-supported movement analysis in children with DCD. Furthermore, previous studies have frequently been limited by relatively small sample sizes, absence of standardized parent training, limited objective biomechanical outcome measures, or lack of randomized controlled designs. Consequently, this study seeks to address an important gap by combining family-centered rehabilitation with objective AI-assisted movement analysis within a rigorously designed randomized controlled trial (Melnyk et al., 2004; Polatajko and Mandich, 2004; Prinsen et al., 2018; Hullumani and Qureshi, 2026; Qureshi et al., 2024).

One of the major strengths of the proposed intervention is the integration of Google MediaPipe Pose, a markerless motion-analysis framework capable of estimating body landmarks from conventional smartphone videos. This technology enables objective quantification of clinically relevant movement characteristics, including joint kinematics, movement symmetry, coordination, movement smoothness, movement accuracy, movement variability, movement speed, and task-completion time. These objective metrics may complement conventional clinical outcome measures by supporting individualized progression, remote monitoring, and data-driven clinical decision-making while reducing dependence on frequent hospital visits. Previous rehabilitation studies have demonstrated encouraging validity and reliability of markerless motion-analysis systems, although evidence specifically involving children with DCD remains limited. Accordingly, AI-derived metrics in the present study will supplement, rather than replace, standardized clinical assessment (Wagh et al., 2025; Latreche et al., 2023; Lam et al., 2023).

Although Google MediaPipe Pose offers a relatively inexpensive and scalable approach for markerless movement analysis, several technical limitations should be acknowledged. Accuracy may be influenced by lighting conditions, camera position, clothing, partial body occlusion, rapid multi-planar movements, background complexity, and smartphone camera quality. To minimize these challenges, the present protocol incorporates standardized video-acquisition procedures, predefined quality-control criteria, automated preprocessing, repeat-recording protocols, and periodic technical monitoring. These measures are expected to improve the reliability and reproducibility of AI-derived movement metrics while maintaining the feasibility of home-based rehabilitation.

Successful implementation of the intervention also depends on active parental participation. Completing approximately 60 home-based sessions of 30–45 min over 12 weeks may impose a considerable time commitment for families, particularly those balancing work, school, and caregiving responsibilities. Furthermore, effective participation requires access to a compatible smartphone, adequate internet connectivity for video transfer or tele-supervision, and sufficient digital literacy to record and upload videos correctly. To address these challenges, parents will receive standardized competency-based training, detailed intervention manuals, demonstration videos, tele-supervision, weekly video review, adherence monitoring, and ongoing technical support. Where internet connectivity is limited, offline recording with subsequent uploading during hospital visits or when connectivity becomes available may reduce implementation barriers. Although the AI platform itself is freely available, wider implementation will require consideration of clinician training, secure data storage, technical support, and overall cost-effectiveness before large-scale adoption.

Privacy and security of participant video recordings also represent important ethical considerations. All videos will be anonymized using coded study identifiers, securely stored on password-protected institutional servers with restricted access, and managed in accordance with institutional ethics and data-protection policies. Personally identifiable information will not be included in video file names, and recordings will not be shared outside the approved research team without appropriate consent.

Despite the strengths of the proposed protocol, several limitations should be acknowledged. The study will be conducted at a single tertiary-care teaching hospital in India, which may limit the generalisability of findings to other healthcare systems, socioeconomic settings, or cultural contexts. Differences in parental involvement, rehabilitation infrastructure, technology availability, and digital literacy may influence implementation in other regions. In addition, because parents and treating therapists cannot be blinded to group allocation, some degree of performance bias may be unavoidable despite assessor blinding and standardized intervention protocols. Variability in parental adherence, home environments, participant motivation, and technological factors may also influence intervention outcomes. Nevertheless, these challenges reflect real-world implementation conditions and therefore enhance the ecological validity of the study.

If demonstrated to be effective, this intervention has the potential to provide an accessible, scalable, and family-centered rehabilitation model that combines conventional physiotherapy with objective AI-assisted movement monitoring. Such an approach may improve motor proficiency, movement quality, and participation in sports while reducing barriers associated with frequent hospital-based rehabilitation. Future multicentre randomized trials should evaluate long-term effectiveness, cost-effectiveness, implementation fidelity, digital accessibility, and cross-cultural applicability before widespread integration into pediatric rehabilitation practice, school-based physical activity programmes, and community health services.
