# Supplementary material for: Development, validation and effectiveness of a parents led neuromotor training module with AI-based movement analysis for improving motor proficiency and sports participation in children with developmental coordination disorder: a protocol for an assessor-blinded randomized superiority controlled trial
Source: Front Hum Neurosci. 2026 Sep 11;20:1931622. doi: 10.3389/fnhum.2026.1931622 (PMC13628172; doi:10.3389/fnhum.2026.1931622)
Supplement: Supplementary file 1 [file Data_Sheet_1.pdf]

1. Parent Education Module

1.1 Overview

The Parent Education Module is the first component of the SMART-MOVE DCD intervention. It is designed to equip parents or primary caregivers with the knowledge, practical skills, and confidence required to safely and effectively deliver a structured home-based neuromotor training programme. The module emphasizes family-centred care by empowering parents to actively participate in their child's rehabilitation while maintaining treatment fidelity through standardized instruction and ongoing therapist supervision.

1.2 Aim

The Parent Education Module aims to:

- Improve parents' understanding of Developmental Coordination Disorder (DCD).
- Explain the importance of early intervention and continuous home-based rehabilitation.
- Train parents to perform neuromotor and sports-specific exercises correctly.
- Standardize intervention delivery across all participants.
- Educate parents on safe exercise supervision and progression.
- Train parents in recording standardized videos for AI-based movement analysis.
- Promote adherence and long-term engagement throughout the intervention.

1.3 Learning Objectives

At the end of the training programme, parents should be able to:

1. Describe the characteristics and functional impact of DCD.
2. Explain the principles of motor learning and task-oriented rehabilitation.
3. Demonstrate all prescribed exercises correctly.
4. Apply appropriate verbal cueing and positive reinforcement.
5. Identify unsafe movement patterns and stop exercises when necessary.
6. Record high-quality videos according to the standardized AI acquisition protocol.
7. Complete the home exercise logbook accurately.
8. Understand progression and regression criteria for exercise difficulty.

9. Communicate effectively with the supervising physiotherapist regarding the child's progress.

1.4 Training Schedule

Session	Topic	Duration	Mode
Session 1	Introduction to DCD, family-centred rehabilitation, and programme overview	60 minutes	Face-to-face
Session 2	Demonstration and supervised practice of neuromotor exercises	60 minutes	Face-to-face
Session 3	Sports-specific training, AI video recording procedures, safety precautions, and competency assessment	60 minutes	Face-to-face

Total Duration: Approximately **3 hours** delivered before initiation of the intervention.

1.5 Educational Content

Module 1: Understanding Developmental Coordination Disorder

Topics covered include:

- Definition and clinical characteristics of DCD.
- Common motor difficulties.
- Functional limitations in daily living.
- Effects on school participation and sports.
- Importance of early intervention.
- Role of parents in rehabilitation.

Module 2: Principles of Home-Based Rehabilitation

Topics include:

- Family-centred rehabilitation.
- Motor learning principles.
- Repetition and task-specific practice.
- Importance of consistency.
- Goal-oriented intervention.

- Positive reinforcement.

Module 3: Neuromotor and Sports Training

Parents receive instruction on:

- Balance training.
- Coordination exercises.
- Agility drills.
- Gross motor activities.
- Fine motor training.
- Sports-specific functional tasks.

Each exercise is demonstrated by the physiotherapist, followed by supervised parent practice.

Module 4: AI-Based Movement Analysis

Parents are trained to:

- Position the camera correctly.
- Record standardized videos.
- Maintain appropriate lighting and background.
- Ensure the child remains fully visible.
- Upload videos according to study procedures.
- Interpret therapist feedback derived from AI analysis.

Module 5: Safety and Monitoring

Topics include:

- Warm-up and cool-down procedures.
- Safe exercise supervision.
- Recognizing fatigue or discomfort.
- Fall prevention.
- When to stop the session.
- Documentation of adverse events.

1.6 Educational Materials Provided

Each parent will receive:

- SMART-MOVE DCD Parent Manual.
- Illustrated exercise booklet.
- Home exercise logbook.
- AI video-recording guide.
- Weekly intervention schedule.
- Safety instruction sheet.
- Therapist contact information.

1.7 Training Methods

The programme uses multiple teaching strategies, including:

- Interactive lectures.
- Live demonstrations by the physiotherapist.
- Supervised return demonstrations by parents.
- Video-assisted learning.
- Printed educational materials.
- Question-and-answer sessions.

1.8 Parent Competency Assessment

Before beginning home-based intervention, each parent must complete a competency assessment conducted by the supervising physiotherapist.

Parents will be evaluated on:

- Knowledge of DCD.
- Correct exercise demonstration.
- Safe handling techniques.
- Verbal cueing.
- AI video-recording procedure.
- Completion of documentation.

Parents must achieve **at least 90% competency** before being certified to deliver the intervention independently. Parents who do not meet this criterion will receive additional training and reassessment.

1.9 Ongoing Support

Throughout the 12-week intervention, parents will receive:

- Weekly therapist supervision.
- Review of AI-generated movement reports.
- Feedback on exercise performance.
- Guidance regarding exercise progression or modification.
- Telephone or video consultations when required.

1.10 Expected Outcomes

Following completion of the Parent Education Module, parents are expected to:

- Demonstrate competence in delivering the intervention.
- Maintain treatment fidelity throughout the study.
- Ensure safe and consistent implementation of home-based rehabilitation.
- Promote active participation and adherence by the child.
- Facilitate accurate AI-based movement assessment through standardized video recordings.

2. Neuromotor & Sports Training Module

2.1 Overview

The Neuromotor and Sports Training Module is the core therapeutic component of the SMART-MOVE DCD intervention. The programme is designed to improve motor proficiency, postural control, coordination, agility, fine motor skills, functional mobility, and sports participation in children with Developmental Coordination Disorder (DCD). The intervention follows principles of task-oriented training, motor learning, graded progression, repetitive practice, and family-centred rehabilitation. Parents serve as the primary facilitators under the supervision of a pediatric physiotherapist.

The programme consists of four integrated components:

1. Balance and Coordination Training
2. Agility Training
3. Sports-Specific Skill Training
4. Fine Motor Skill Training

Each component is progressively advanced throughout the 12-week intervention according to predefined progression criteria.

2.2 Programme Schedule

**Item**	**Description**
Programme duration	12 weeks
Frequency	5 sessions/week
Session duration	30–45 minutes
Total supervised sessions	60 sessions
Delivered by	Parent under therapist supervision
Therapist review	Weekly
AI review	Weekly

2.3 Standard Session Structure

Each home-based training session follows the same standardized sequence

Activity	Duration
Warm-up	5 minutes
Balance & Coordination Training	10 minutes
Agility Training	8 minutes
Sports-Specific Training	10 minutes
Fine Motor Training	7 minutes
Cool-down	5 minutes

2.4 Balance and Coordination Training

Aim

To improve postural stability, weight transfer, body awareness, bilateral coordination, and dynamic balance.

Activities

- Static standing balance
- Tandem standing
- Single-leg standing
- Weight-shifting activities
- Heel-to-toe walking
- Straight-line walking
- Obstacle stepping
- Side stepping
- Dynamic reaching
- Functional balance games

Dosage

- 3 sets per activity
- 8–12 repetitions
- 30–60 seconds rest
- Approximately 10 minutes/session

Progression

- Narrower base of support
- Reduced upper-limb support
- Increased task duration
- Eyes closed (where appropriate)
- Foam surface
- Dual-task performance

Regression

- Wider base of support
- Hand support
- Reduced repetitions
- Therapist/parent assistance

2.5 Agility Training

Aim

To improve movement speed, directional changes, reaction time, and dynamic motor control.

Activities

- Shuttle walking
- Shuttle running
- Cone weaving
- Side shuffling
- Zig-zag walking
- Obstacle course
- Ladder drills
- Directional stepping
- Reaction games

Dosage

- 3 sets
- 20–30 seconds/activity
- 60 seconds rest
- Approximately 8 minutes/session

Progression

- Increase speed
- Reduce completion time
- Increase obstacle complexity
- Add dual-task activities

Regression

- Reduce speed
- Simplify pathway
- Increase rest periods

2.6 Sports-Specific Skill Training

Aim

To enhance sports participation by improving functional motor skills required during recreational and school-based physical activities.

Activities

- Throwing
- Catching
- Kicking
- Ball bouncing
- Ball control
- Target throwing
- Jumping
- Running

- Relay games
- Modified sport-specific games

Dosage

- 3 sets
- 8–15 repetitions
- Approximately 10 minutes/session

Progression

- Increase throwing distance
- Smaller targets
- Faster movement
- Reduced external cues
- Multi-directional activities

Regression

- Larger targets
- Shorter distances
- Stationary practice
- Parent assistance

2.7 Fine Motor Skill Training

Aim

To improve hand dexterity, manual coordination, hand–eye coordination, and precision movements.

Activities

- Pegboard activities
- Bead threading

- Coin transfer
- Buttoning
- Cloth peg activities
- Drawing
- Cutting
- Object manipulation
- Hand strengthening
- Bilateral hand coordination tasks

Dosage

- 2–3 sets
- 5–10 repetitions/task
- Approximately 7 minutes/session

Progression

- Smaller objects
- Increased speed
- Reduced visual guidance
- Bilateral tasks
- Functional task integration

Regression

- Larger objects
- Reduced repetitions
- Simplified tasks

2.8 Weekly Progression Plan

Weeks	Training Focus	Expected Progression
1–2	Familiarization and basic motor control	Static balance, simple coordination, basic sports tasks
3–4	Dynamic balance and locomotor skills	Walking patterns, obstacle negotiation, throwing and catching

5–6	Agility and movement efficiency	Directional changes, shuttle activities, ball control
7–8	Complex coordination	Dual-task activities, advanced balance, reaction tasks
9–10	Sports integration	Sport-specific drills, dynamic running, jumping, kicking
11–12	Functional participation	Community-based activities, independent task performance, advanced sports participation

2.9 Progression Criteria

Exercises will be progressed only when the child:

- Completes at least **80% of repetitions correctly**.
- Demonstrates safe movement patterns.
- Maintains balance throughout the activity.
- Requires minimal verbal cueing.
- Reports no pain or excessive fatigue.
- Successfully completes the same activity during two consecutive sessions.

Progression may include:

- Increasing repetitions.
- Increasing duration.
- Increasing movement speed.
- Reducing external support.
- Increasing task complexity.
- Introducing dual-task conditions.
- Incorporating sport-specific functional tasks.

2.10 Regression Criteria

Exercise difficulty will be reduced if the child:

- Completes fewer than **60% of repetitions correctly**.

- Demonstrates repeated loss of balance.
- Shows excessive compensatory movements.
- Reports pain or discomfort.
- Experiences excessive fatigue.
- Is unable to complete the prescribed task safely.

Regression strategies include:

- Simplifying movement patterns.
- Increasing external support.
- Reducing repetitions.
- Increasing rest periods.
- Returning to the previous progression level.

2.11 Home Programme

Parents will supervise five sessions per week using the standardized intervention manual. All completed sessions will be documented in the Home Exercise Logbook. Weekly videos of selected motor tasks will be submitted for AI-based movement analysis and therapist review. Feedback from these assessments will guide progression while maintaining adherence to the standardized protocol.

3. AI-Based Movement Analysis

3.1 Overview

Artificial intelligence (AI)-based movement analysis will be integrated into the SMART-MOVE DCD intervention to provide objective, quantitative, and reproducible assessment of movement quality during home-based rehabilitation. The AI system will support physiotherapists by monitoring motor performance, identifying movement deviations, tracking longitudinal progress, and facilitating individualized feedback for progression of the intervention.

The movement analysis system will utilize **Google MediaPipe Pose**, an open-source, real-time, markerless human pose estimation framework capable of detecting **33 anatomical body landmarks** from RGB videos recorded using standard smartphone cameras. The AI system will be used exclusively as an assessment and feedback tool and will not replace clinical decision-making by the treating physiotherapist.

3.2 Standardized Video Acquisition Protocol

To ensure consistency across participants, all videos will be recorded using a standardized acquisition protocol.

**Parameter**	**Standard**
Recording device	Android or iOS smartphone
Camera	Rear camera preferred
Minimum resolution	1920 × 1080 pixels (1080p)
Frame rate	Minimum 30 frames/second
Orientation	Landscape
Recording distance	2.5–3.0 metres
Camera height	Approximately pelvic level of the child
Camera angle	Perpendicular to the movement plane (frontal or sagittal)
Background	Plain, uncluttered, non-reflective
Lighting	Uniform indoor lighting (approximately ≥300 lux)
Clothing	Tight-fitting clothing exposing body contours
Footwear	Standard athletic shoes or barefoot (consistent across assessments)

Recording duration	30–60 seconds per motor task

Each participant will perform standardized motor tasks during baseline, Week 6, Week 12, and Week 24 assessments under identical recording conditions.

3.3 Video Recording Procedure

Parents will receive standardized training before initiating home recordings.

The recording procedure will include:

1. Position the smartphone on a stable tripod.
2. Ensure the child's entire body is visible throughout the recording.
3. Maintain the prescribed recording distance (2.5–3.0 m).
4. Record the predefined motor task without camera movement.
5. Save the video using the participant identification code.
6. Upload the recording through the designated secure platform within 24 hours.

The physiotherapist will verify video quality before AI analysis.

3.4 AI Processing Workflow

The AI analysis will follow the workflow below:

Video Recording → MediaPipe Pose Detection → Landmark Extraction (33 body landmarks) → Data Quality Check → Signal Processing → Joint Kinematic Analysis → AI-Derived Outcome Measures → Therapist Review → Individualized Feedback

3.5 Preprocessing of Landmark Data

Raw landmark coordinates obtained from MediaPipe Pose will undergo preprocessing before biomechanical analysis.

The preprocessing pipeline will include:

- Removal of low-confidence landmarks.
- Fourth-order zero-phase Butterworth low-pass filtering (cut-off frequency: 6 Hz) to reduce measurement noise.
- Cubic spline interpolation for short periods of missing landmark detection (<5 consecutive frames).
- Time normalization of repeated movement cycles when applicable.
- Calculation of joint angles using two-dimensional body landmark coordinates.

Videos containing excessive missing data (>5% of total frames) will not undergo interpolation and will instead be reacquired.

3.6 Quality Control Procedures

Each recording will undergo automated and manual quality assessment before analysis.

A recording will be accepted only when:

- ≥95% of frames contain valid landmark detection.
- Entire body remains visible throughout the recording.
- No major body-segment occlusion is present.
- Camera remains stationary.
- Lighting conditions are adequate.
- Image quality is sufficient for accurate landmark identification.

Videos will be rejected and repeated if any of the following occur:

- Excessive motion blur.
- Poor illumination.
- Partial body visibility.
- Camera movement.
- Landmark tracking failure.
- Background interference.

3.7 Reliability Monitoring

To evaluate measurement reliability, the following indices will be monitored:

- Percentage of valid landmark frames.
- Mean landmark confidence score.
- Intraclass Correlation Coefficient (ICC) for repeated recordings.
- Root Mean Square Error (RMSE) between repeated trials.

An ICC value ≥ 0.80 will be considered indicative of acceptable reliability for repeated assessments.

3.8 AI-Derived Outcome Measures

The AI system will automatically calculate the following variables:

1. Movement Symmetry Index

Measures bilateral symmetry during task performance by comparing left and right limb movements.

Higher values indicate greater movement symmetry.

2. Coordination Index

Represents temporal coordination between adjacent body segments based on synchronized joint motion.

Higher scores indicate better intersegmental coordination.

3. Movement Accuracy

Calculated as the deviation between the participant's movement trajectory and the standardized reference movement demonstrated during training.

Lower values indicate greater movement accuracy.

4. Movement Smoothness

Quantified using normalized jerk and trajectory smoothness.

Higher smoothness indicates improved motor control.

5. Movement Speed

Average linear velocity during task execution.

Reported in metres per second (m/s).

6. Joint Kinematics

Joint angles and range of motion will be calculated for:

- Shoulder
- Elbow
- Hip
- Knee
- Ankle
- Trunk

7. Movement Variability

Standard deviation of repeated movement trajectories.

Lower variability represents greater motor consistency.

8. Task Completion Time

Time required to complete each standardized motor task.

Reported in seconds.

3.9 AI-Assisted Feedback

Following analysis, AI-generated reports will be reviewed by the treating physiotherapist.

Feedback will include recommendations regarding:

- Movement symmetry
- Coordination deficits
- Balance performance

- Joint movement quality
- Progression or regression of exercise difficulty
- Home exercise modification

Only the physiotherapist will make final clinical decisions regarding intervention progression.

3.10 Limitations of AI-Based Analysis

The accuracy of markerless pose estimation may be influenced by environmental and technical factors, including inadequate lighting, camera positioning, motion blur, loose clothing, body-segment occlusion, and rapid out-of-plane movements. These limitations will be minimized through standardized recording procedures, parent training, predefined quality-control criteria, and repeat recording of videos that fail to meet the required quality standards.

4. Standard Session Structure

Activity	Duration
Warm-up	5 min
Neuromotor Training	15 min
Sports Training	10 min
Fine Motor	10 min
Cool Down	5 min

Total- 45 min

5. Weekly Progression

Weeks	Focus
1–2	Basic balance
3–4	Dynamic balance
5–6	Agility
7–8	Sports
9–10	Complex tasks
11–12	Community participation

6. Progression Criteria

Progress if,

- 80% correct
- No pain
- Good balance
- Parent competency

7. Parent Monitoring

- Weekly
- Video upload
- Logbook
- Phone call

- AI review

8. Safety

Stop exercise if.

- Pain
- Fatigue
- Fall
- Distress

Appendix A- Parent Competency Checklist

Purpose: This checklist is used to evaluate whether parents/caregivers have achieved the minimum competency required to independently deliver the **SMART-MOVE DCD Parent-Led Neuromotor Training Programme** at home. Competency is assessed immediately after the parent training programme and before commencement of the intervention.

Instructions to the Assessor: Observe the parent while they demonstrate the intervention procedures using a child or simulated practice. Mark the appropriate score for each competency item, **Scoring System:**

- **2 = Competent** (Performs independently and correctly)
- **1 = Partially Competent** (Performs with minor errors or requires verbal prompting)
- **0 = Not Competent** (Unable to perform or performs incorrectly)

Section A. Knowledge of Developmental Coordination Disorder (DCD)

No.	Competency Item	0	1	2
1	Explains the basic characteristics of Developmental Coordination Disorder (DCD).	☐	☐	☐
2	Describes the purpose and goals of the SMART-MOVE DCD programme.	☐	☐	☐
3	Understands the importance of regular home-based practice and adherence.	☐	☐	☐

Section B. Parent Education and Communication Skills

No.	Competency Item	0	1	2
4	Provides clear verbal instructions appropriate for the child.	☐	☐	☐
5	Demonstrates positive encouragement and motivation throughout the session.	☐	☐	☐
6	Provides constructive feedback without criticism or frustration.	☐	☐	☐

Section C. Neuromotor and Sports Training Skills

No.	Competency Item	0	1	2
7	Correctly demonstrates balance exercises.	☐	☐	☐
8	Correctly demonstrates coordination exercises.	☐	☐	☐
9	Correctly demonstrates agility exercises.	☐	☐	☐
10	Correctly demonstrates sports-specific training activities.	☐	☐	☐
11	Correctly demonstrates fine motor training activities.	☐	☐	☐
12	Maintains the prescribed exercise dosage (sets, repetitions, duration, and rest intervals).	☐	☐	☐

Section D. Safety and Child Handling

No.	Competency Item	0	1	2
13	Maintains a safe exercise environment free of hazards.	☐	☐	☐
14	Demonstrates safe guarding and supervision throughout exercises.	☐	☐	☐
15	Recognizes signs of fatigue, pain, or distress requiring exercise modification or termination.	☐	☐	☐

Section E. AI-Based Movement Analysis

No.	Competency Item	0	1	2
16	Correctly positions the smartphone camera according to the protocol.	☐	☐	☐
17	Records videos with appropriate lighting, background, and full-body visibility.	☐	☐	☐
18	Correctly uploads or submits videos for AI-based movement analysis.	☐	☐	☐
19	Understands how AI-generated feedback will be used to guide exercise progression.	☐	☐	☐

Section F. Documentation and Adherence

No.	Competency Item	0	1	2
20	Correctly completes the daily home exercise logbook.	☐	☐	☐
21	Records completed sessions accurately and consistently.	☐	☐	☐

22	Understands the procedure for reporting missed sessions or adverse events.	☐	☐	☐

Scoring

Classification	Total Score
Maximum Score	**44**
Minimum Competency Required	**≥40/44 (≥90%)**
Below Competency	**<40/44**

Interpretation:

- **40–44:** Competent to independently deliver the SMART-MOVE DCD programme.
- **30–39:** Requires additional supervised training before certification.
- **<30:** Not competent; complete retraining and reassessment required.

Assessor's Recommendation

☐ Competent – Approved to commence the home-based intervention.

☐ Requires additional supervised training.

☐ Requires complete retraining before reassessment.

Assessor Details

Parent/Caregiver Name: ______________________________

Child Study ID: ______________________________

Date of Assessment: ____ / ____ / ______

Assessor (Physiotherapist): ______________________________

Signature: ______________________________

Appendix B- SMART-MOVE DCD Daily Home Exercise Logbook

Participant Information,

Variable	Details
Participant ID	____________________________
Parent/Caregiver Name	____________________________
Child Age	_________ Years
Week Number	_________
Therapist Name	____________________________

Daily Home Exercise Record

Date	Session Completed (✓/✗)	Parent Education Reviewed (Y/N)	Balance & Coordination	Agility Training	Sports Skill Training	Fine Motor Skills	Duration (Minutes)	Child Participation (Excellent/Good/Fair/Poor)	Difficulty Level (Easy/Moderate/Hard)	Pain/Fatigue (Y/N)
Monday										
Tuesday										
Wednesday										
Thursday										
Friday										

Weekly Exercise Summary

Component	Planned Sessions	Completed Sessions	Percentage Completed
Balance & Coordination	5	____	____ %
Agility Training	5	____	____ %
Sports Skill Training	5	____	____ %
Fine Motor Skills	5	____	____ %
AI Video Submission	1	____	____ %

Therapist Review (Completed During Weekly Follow-up)

Item	Yes	No	Comments
Exercise programme completed as prescribed	☐	☐	
Correct exercise technique observed	☐	☐	
Parent followed intervention protocol	☐	☐	
AI video quality acceptable	☐	☐	
Exercise progression required	☐	☐	
Retraining required	☐	☐	
Adverse events reported	☐	☐	

Weekly Adherence Calculation

$$\text{Adherence (\%)} = \frac{\text{Number of sessions completed}}{\text{Number of prescribed sessions}} \times 100$$

Adherence Classification

Adherence (%)	Interpretation
≥ 90%	Excellent
80–89%	Good
60–79%	Moderate
< 60%	Poor (Retraining Required)

Safety Checklist

Please tick if any of the following occurred during the week:

☐ Child experienced pain during exercise

☐ Child experienced excessive fatigue

☐ Child lost balance or fell

☐ Exercise session was stopped

☐ Technical problem with AI recording

☐ Parent required additional guidance

☐ No problems encountered

Instructions for Parents

1. Complete one log after each home session.
2. Record the duration of the session and all activities performed.
3. Submit one AI recording each week according to the study protocol.
4. Contact the study physiotherapist if your child experiences pain, dizziness, excessive fatigue, or any adverse event.
5. Bring the completed logbook to the weekly follow-up visit or upload it electronically if instructed.

Appendix C- Therapist Fidelity Monitoring Checklist

Therapist Fidelity Assessment Form

Participant ID: _______________________

Parent/Caregiver Name: _______________________

Week of Intervention: _______________________

Therapist Name: _______________________

Date of Review: _______________________

Section A. Parent Training and Competency

Item	Yes	No	Comments
Parent attended the initial training programme	☐	☐	
Parent demonstrated correct understanding of DCD	☐	☐	
Parent demonstrated all prescribed exercises correctly	☐	☐	
Parent completed competency assessment successfully	☐	☐	
Parent received intervention manual and educational materials	☐	☐	

Section B. Intervention Delivery

Item	Yes	No	Comments
Exercises performed according to protocol	☐	☐	
Correct sequence of intervention followed	☐	☐	
Correct dosage (sets/repetitions) maintained	☐	☐	
Recommended session duration maintained (30–45 min)	☐	☐	
Five sessions completed during the week	☐	☐	

Section C. Exercise Quality

Item	Yes	No	Comments
Balance exercises performed correctly	☐	☐	

Coordination exercises performed correctly	☐	☐	
Agility exercises performed correctly	☐	☐	
Sports-specific activities completed	☐	☐	
Fine motor activities completed	☐	☐	

Section D. AI-Based Movement Analysis

Item	Yes	No	Comments
Weekly video submitted	☐	☐	
Camera positioned correctly	☐	☐	
Recording quality acceptable	☐	☐	
AI analysis completed successfully	☐	☐	
AI feedback communicated to parent	☐	☐	

Section E. Safety Monitoring

Item	Yes	No	Comments
Child completed exercises safely	☐	☐	
No falls or injuries reported	☐	☐	
No excessive fatigue observed	☐	☐	
Parent followed safety instructions	☐	☐	
No adverse events occurred	☐	☐	

Section F. Parent Adherence

Item	Yes	No	Comments
Daily logbook completed	☐	☐	
Home programme followed	☐	☐	
Parent remained motivated	☐	☐	
Child actively participated	☐	☐	

Weekly review completed	☐	☐	

Section G. Protocol Deviations

Tick all that apply.

☐ Missed sessions

☐ Incorrect exercise technique

☐ Incorrect exercise dosage

☐ AI recording not submitted

☐ Poor video quality

☐ Parent unable to perform exercises

☐ Child illness

☐ Technical issues

☐ Other: ____

Section H. Overall Fidelity Rating

Criterion	Rating
Parent competency	□ Excellent □ Good □ Fair □ Poor
Exercise delivery	□ Excellent □ Good □ Fair □ Poor
AI implementation	□ Excellent □ Good □ Fair □ Poor
Protocol adherence	□ Excellent □ Good □ Fair □ Poor
Overall intervention fidelity	□ Excellent □ Good □ Fair □ Poor

Therapist Declaration

I confirm that the intervention was reviewed according to the SMART-MOVE DCD intervention protocol and that the above information accurately reflects the participant's intervention fidelity.

Therapist Name: ____

Signature: ________________________

Date: ________________________

Appendix D- AI Video Recording Guide

Purpose

This guide provides standardized instructions for recording videos for AI-based movement analysis using **Google MediaPipe Pose**. Adherence to these procedures will ensure high-quality recordings, reliable landmark detection, and consistent movement analysis across participants.

1. Equipment Required

Equipment	Specification
Smartphone	Android or iOS smartphone with HD camera
Camera Resolution	Minimum 1920 × 1080 pixels (1080p)
Frame Rate	Minimum 30 frames/second (fps)
Tripod	Adjustable tripod or stable stand
Internet	Required for secure video upload
Recording Space	Minimum 3 m × 3 m

2. Recording Environment

The recording environment should meet the following conditions:

- Plain, non-reflective background (preferably white or light grey)
- Uniform lighting without strong shadows or backlighting
- Well-lit room (approximately ≥300 lux)
- Quiet environment free from distractions
- Floor should be flat, dry, and free of obstacles
- Only the child should be visible during recording

3. Participant Preparation

Before recording:

- Wear **tight-fitting, comfortable clothing** that allows visualization of body movements.
- Avoid loose jackets, long dresses, or oversized clothing.
- Wear the same footwear (or remain barefoot if specified) throughout the study.
- Remove hats, scarves, or accessories that may obscure body landmarks.
- Tie long hair away from the face and shoulders if necessary.

4. Camera Position

Parameter	Standard
Distance from participant	2.5–3.0 metres
Camera height	Approximately at pelvis level
Camera orientation	Landscape mode
Camera stability	Mounted on tripod or stable surface
Zoom	No digital zoom
Camera movement	None during recording

5. Camera View

Frontal Tasks

The camera should face the participant directly.

Camera
|
Participant

Side-View Tasks

The camera should be positioned at 90° to the participant.

Camera → Participant

6. Recording Procedure

1. Ensure the participant's entire body is visible throughout the recording.
2. Begin recording 2–3 seconds before movement initiation.
3. Perform the task exactly as instructed.
4. Continue recording until 2–3 seconds after task completion.
5. Record **three repetitions** of each task.
6. Do not stop recording between repetitions unless instructed.

7. Standardized Motor Tasks

Videos should be recorded for the following activities:

- Static standing balance
- Tandem walking
- Single-leg stance
- Walking
- Running
- Throwing
- Catching
- Jumping
- Agility drill
- Fine motor task (where applicable)

8. Video Quality Checklist

Before submitting the recording, confirm:

☐ Entire body visible

☐ Face visible (if required)

☐ Adequate lighting

☐ No shadows covering the body

☐ Camera stable

☐ Background clear

☐ No other person visible

☐ No excessive noise or distractions

☐ Correct task completed

☐ Three repetitions recorded

9. Video File Naming

Videos should be labelled as follows:

Participant ID_Task_Week_Date

Example: DCD021_Balance_Wk06_2026-08-15.mp4

10. Video Submission Schedule

Assessment	Time Point
Baseline	Week 0
Interim	Week 6
Post-intervention	Week 12
Follow-up	Week 24

Additional weekly videos may be requested for monitoring and feedback.

11. Quality-Control Criteria

Videos will be reviewed before AI analysis.

A recording will be **accepted** if:

- ≥95% of frames contain valid landmark detection
- Entire body remains visible throughout the recording
- Camera remains stable
- Lighting is adequate
- Task is completed correctly

12. Rejection Criteria

A recording will be repeated if any of the following occur:

- Poor lighting
- Excessive motion blur
- Camera movement
- Body segments obscured
- Participant leaves the camera field
- Incorrect camera angle
- Landmark tracking failure for >5 consecutive frames
- Incorrect task performed

13. AI Processing Workflow

Video Recording

↓

Quality Check

↓

MediaPipe Pose

(33 Landmark Detection)

↓

Landmark Confidence Check

↓

Filtering and Data Processing

↓

Calculation of AI Metrics

• Symmetry Index

• Coordination Index

• Movement Accuracy

• Movement Smoothness

• Movement Speed

• Joint Kinematics

↓

Therapist Review

↓

Individualized Feedback to Parents

14. Troubleshooting Guide

Problem	Possible Cause	Solution
Child not fully visible	Camera too close	Move camera to 2.5–3 m
Dark image	Poor lighting	Increase room lighting
Blurred movement	Camera shaking	Use tripod
Landmark tracking failure	Loose clothing or occlusion	Use fitted clothing and repeat recording
Incomplete recording	Camera stopped early	Re-record the task

Incorrect view	Camera angle incorrect	Position camera according to protocol

15. Parent Confirmation Checklist

Before uploading each recording, parents should confirm:

☐ Recording followed the protocol.

☐ Camera distance was correct.

☐ Camera remained stable.

☐ Child performed the correct task.

☐ Entire body remained visible.

☐ Lighting was adequate.

☐ Video file was correctly named.

☐ Video uploaded successfully
