# Supplementary material for: Development, validation and effectiveness of a parents led neuromotor training module with AI-based movement analysis for improving motor proficiency and sports participation in children with developmental coordination disorder: a protocol for an assessor-blinded randomized superiority controlled trial
Source: Front Hum Neurosci. 2026 Sep 11;20:1931622. doi: 10.3389/fnhum.2026.1931622 (PMC13628172; doi:10.3389/fnhum.2026.1931622)
Supplement: Supplementary file 2 [file Data_Sheet_2.pdf]

Parent Competency Assessment Checklist

Participant code: __________

Parent/caregiver code: __________

Assessor: __________

Date: __________

No.	Competency item	0	1	2
1	Explains the purpose, frequency, duration, and sequence of the home programme	☐	☐	☐
2	Prepares a safe, adequately lit, and obstacle-free exercise area	☐	☐	☐
3	Positions the child correctly and provides appropriate physical assistance	☐	☐	☐
4	Demonstrates the prescribed balance exercises correctly	☐	☐	☐
5	Demonstrates coordination and bilateral motor activities correctly	☐	☐	☐
6	Demonstrates task-oriented and sports-related activities according to the intervention manual	☐	☐	☐
7	Provides clear verbal instructions, demonstrations, encouragement, and corrective feedback	☐	☐	☐
8	Follows the prescribed repetitions, duration, rest intervals, and progression criteria	☐	☐	☐
9	Recognizes fatigue, pain, dizziness, distress, loss of balance, or other adverse responses and responds appropriately	☐	☐	☐
10	Records videos using the standardized camera position, angle, distance, lighting, clothing, and framing requirements	☐	☐	☐
11	Completes the adherence logbook accurately and reports missed sessions or difficulties	☐	☐	☐
12	Follows safety, confidentiality, secure video-upload, and data-privacy procedures	☐	☐	☐

Scoring

- **0:** Unable to perform, performs incorrectly, or creates a safety concern.
- **1:** Performs partially or requires verbal or physical prompting.
- **2:** Performs independently, correctly, and consistently.

Total score: ____ / 24

Competency Criterion

A parent will be considered competent when:

- the total score is **at least 22 out of 24**;
- Items 2, 3, 8, 9, 10, and 12 receive a score of **2**; and
- no activity is performed in a manner that places the child at risk.

Decision:

- ☐ Competent and certified for home intervention
- ☐ Additional training required
- ☐ Reassessment required

Assessor signature: ____________________
